# The Relationship Between Strategic Human Resource Management Practices and the Employment of Vulnerable Workers: A Two-Wave Study Among Employers

**DOI:** 10.1007/s10926-024-10197-9

**Published:** 2024-05-04

**Authors:** Amber Kersten, Marianne van Woerkom, Goedele A. Geuskens, Roland W. B. Blonk

**Affiliations:** 1 Department of Human Resource Studies, School of Social and Behavioral Sciences, Tilburg University, Tilburg, The Netherlands; 2 Healthy Living, Netherlands Organization for Applied Scientific Research (TNO), Leiden, The Netherlands; 3 Optentia Research Focus Area, North-West University, Vanderbijlpark, South Africa

**Keywords:** Inclusion, Human resource management, Vulnerable workers, Employer engagement

## Abstract

**Purpose:**

To improve the inclusion of vulnerable workers in the labor market, employer behavior is key. However, little is known about the effectiveness of strategic Human Resource Management (HRM) practices that employers use to employ vulnerable workers. Therefore, this exploratory study investigates the association between strategic HRM practices (based on social legitimacy, economic rationality and employee well-being) and the actual and intended employment of vulnerable workers in the future.

**Methods:**

In total, 438 organizations included in the Netherlands Employers Work Survey participated in a two-wave study with a nine-month follow-up period. Logistic regression models were used to estimate the relationship between strategic HRM practices (T0) with the employment of vulnerable workers (T1) and intentions to hire vulnerable workers (T1), while controlling for organizational size, sector, and employment of vulnerable workers at baseline.

**Results:**

Employers who applied strategic HRM practices based on social legitimacy (e.g., inclusive mission statement or inclusive recruitment) or economic rationality (e.g., making use of reimbursements, trial placements, or subsidies) at T0 were more likely to employ vulnerable workers and to intend to hire additional vulnerable workers at T1. No significant results were found for practices related to employee well-being.

**Conclusion:**

Since different types of strategic HRM practices contribute to the inclusion of vulnerable workers, employers can build on their strategic priorities and strengths to create inclusive HRM approaches. Future research is needed to study whether these strategic HRM domains also relate to sustainable employment of vulnerable workers.

**Supplementary Information:**

The online version contains supplementary material available at 10.1007/s10926-024-10197-9.

## Introduction

Across the globe, numerous workers are excluded from stable and mainstream employment. These so-called vulnerable workers, such as people with disabilities or with a migration background, are thought to encompass the largest underused potential on the labor market today [1]. Vulnerable workers are prone to unstable employment due to an interaction between personal characteristics (e.g., a disability) and the precarious work context in which they often operate (e.g., temporary employment) [2–5]. Hence, including vulnerable workers in mainstream employment requires interventions that are aimed at both the individual and their work context.

Previous research, however, has largely overlooked the role of employers in promoting participation of vulnerable workers in the labor market [6]. This is an important omission since employers have a substantial influence on the access to sustainable work for vulnerable workers [7], but often do not know how to successfully recruit and retain these workers [8–14]. So far, the literature on Human Resource Management (HRM) practices that targets the employment of vulnerable workers has focused primarily on the importance that employers attach to these practices and on the prediction of hiring intentions [15–17]. Only a few studies have investigated the relationship between HRM practices and the *actual* employment of vulnerable workers, for instance by studying the effect of organizational policies on the inclusion of workers with disabilities [18, 19]. Still, these studies are primarily focused on workers with disabilities and do not include other workers with a distance to the labor market. Hence, more insight is needed into the value of HRM practices for the employment of a broader population of vulnerable workers. This specifically holds for *strategic* HRM [7, 12, 14, 15], which could help to apply a more strategic lens to the relevant societal topic of inclusion of vulnerable workers. Strategic HRM pertains to the idea that organizations are driven by certain strategic goals that underly their business model [20]. Examples of such strategic goals are goals related to social legitimacy, economic rationality or employee well-being. Social legitimacy goals are based on the motivation to generate (shareholder) value by acting in line with fair and ethical principles, economic rationality goals refer to employers striving to be productive and cost-efficient organizations, and employee well-being goals refer to employers striving to maintain happy and healthy workers within their organization [7]. Within this study, we explore to what extent these three types of strategic HRM practices can contribute to the inclusion of vulnerable workers.

To this end, this study answers the following two research questions: (1) How frequently are strategic, inclusive HRM practices applied by employers and to what extent do these practices differ across organizations of different sizes and sectors? (2) To what extent do strategic inclusive HRM practices predict the actual employment of vulnerable workers and the intention to hire vulnerable workers?

By answering these research questions, we contribute to the rehabilitation literature in three different ways. First, we address the scant attention in the rehabilitation literature for strategic HRM practices and their effects on inclusive employer behavior. Previous studies have mostly focused on the effects that beliefs about (hiring) vulnerable workers, workplace characteristics, or motivations have on the hiring of vulnerable groups [e.g., 16], while overlooking the effects of strategic HRM practices. This strategic HRM focus is essential, as this promotes the alignment between the HRM-related topic, in this case the inclusion of vulnerable groups, with the overall goals of the organization, hence increasing the strategic relevance [20]. In addition, most of the literature has focused on predicting the intention to hire vulnerable workers instead of the actual hiring of these workers [15]. However, research has shown that hiring intention may not always lead to actual hiring of vulnerable workers [21]. Therefore, we study both hiring intention and actual employment [16]. Second, we study the often-overlooked employer’s perspective [6] on the inclusion of vulnerable workers by studying employers instead of employees. Since employers decide on the hiring of vulnerable workers [7], it is important to capture their perspective in research. Factors that may drive or hamper their hiring behavior, such as cost-related considerations related to hiring vulnerable workers, may be overlooked in employee-focused research. Third, we study the employment of various groups of vulnerable workers, i.e., people with disabilities, long-term unemployed people, people with a migration background, and low-educated people. Hence, we respond to recent calls to investigate the effectiveness of HRM practices on a wide variety of vulnerable workers [22], instead of people with disabilities who have so far gotten most of the research attention.

## Theoretical Background

The strategic HRM literature distinguishes three types of HRM practices: practices based on social legitimacy, economic rationality, and employee well-being [7, 23]. These HRM practices correspond to the three levels of impact of HRM practices, as distinguished in the Harvard Model [24], on societal well-being, organizational effectiveness and individual well-being. Below, we elaborate on inclusive HRM practices that are related to the strategic domains of social legitimacy, economic rationality and employee well-being and their potential impact on the employment of vulnerable workers and intention to recruit vulnerable workers.

Firstly, social legitimacy-related HRM practices are aimed at making positive, societal impact [7]. These practices build on a relational rationality [25, 26], which allows organizations to generate and demonstrate moral value for society as a whole [27]. An important indicator of a social legitimacy perspective is the mission statement of the organization. An inclusive mission statement is thought to be associated with the hiring of vulnerable workers, since these organizations explicitly demonstrate that they are driven by a motivation to progress labor market inclusion of vulnerable groups [7]. Alongside mission statements, previous studies have shown that organizations seek social legitimacy through the application of inclusive recruitment practices, such as recruitment through specialized agencies or job creation for vulnerable groups [28]. These practices are explicitly aimed at hiring vulnerable groups, which may require alternative recruitment efforts (e.g., working together with specialized agencies), enabling sustainable employment for all [29], and a fair representation of society within the organization [30]. Inclusive recruitment practices that have been found to support the inclusion of vulnerable groups are, for example, job creation, work experience positions, seconding vulnerable workers, or collaborating with other employers for the recruitment of vulnerable groups [17, 28, 31]. These practices are especially important as the vast majority of (inclusive and non-inclusive) employers experiences problems in hiring vulnerable groups [31].

Inclusive HRM practices based on economic rationality focus on sustaining organizational effectiveness by ensuring financial performance and cost minimization [7, 32]. Within the Netherlands, inclusive economic rationality practices include making use of arrangements offered by the government such as wage subsidies, reimbursements for adaptations of the workplace, reimbursements for job coaches, and reimbursement for trial placement. Wage subsidies and wage arrangements can be seen as financial incentives to hire vulnerable workers, whereas accommodative practices, such as reimbursement for job coaches or adaptations of the workplace, support the successful integration of the vulnerable worker. Previous research highlights that some employers are concerned about the costs associated with hiring vulnerable workers [8, 21, 33]. Practices based on economic rationality may reduce these perceived barriers [34, 35].

Lastly, employee well-being practices include practices aimed at ensuring the well-being of the individual employee, such as adapting work hours, offering development opportunities, stimulating job crafting, and adapting the workplace. The well-being HRM domain aims for a win–win situation in which both the well-being and high performance of the employee is achieved [36]. Particularly for vulnerable workers, well-being practices are thought to be important to ensure their sustainable employment [18, 37]. For instance, previous research has shown that adapting work hours, stimulating training, and job crafting are positively related to the employment of vulnerable workers [31].

As part of our exploratory approach, we investigate the relationship between the abovementioned strategic HRM practices related to social legitimacy, economic rationality, and employee well-being on the one hand, and both the current employment of vulnerable workers and the intention to hire these workers in the future on the other hand. Previous research, which builds on the Integrative Model of Behavioral Prediction [38], highlighted that the intention to hire people with a distance to the labor market may differ from the actual hiring of these employees. Whereas employer attitude [8, 21], subjective norms [31], and perceived behavioral control [31] have been found to predict the intention to hire vulnerable workers, the presence of a disability hiring policy was found to be a more reliable predictor of the actual hiring of vulnerable workers [21]. Therefore, we explore not only whether the application of strategic HRM practices leads to the intention to hire vulnerable groups in the future, but also whether these HRM practices result in the (continued) employment of these workers.

## Methods

### Participants and Procedure

Data were collected in September 2021 (T0), as a part of the Netherlands Employers Work Survey (NEWS), which is a two-yearly survey that strives to provide insights into the employment practices of a representative sample of Dutch employers [39]. In total, 24,983 employers, who were randomly selected from the Netherlands National Job Information System, were invited to participate in the 2021 measurement of NEWS. The selected sample of directors and (HR) managers received an announcement letter, which contained a unique code that allowed the participant to open the survey. In total, 4,791 participants filled in the entire survey (response rate of 19.2%). 1,367 participants indicated to be willing to fill in a second survey. In June 2022 (T1) 994 respondents were invited to participate in this follow-up survey and received a questionnaire (73.7%). The remaining 373 participants, who had reported nightshift-work were not invited as they participated in another study on nightshift-work. Of the 994 persons invited, fifteen emails bounced, resulting in a sample of 979 respondents for the second measurement (T1). A total of 438 respondents filled in the second measurement (44.1%). No differences between respondents and non-respondents were found, except for respondents significantly more often working in non-profit organizations (see Appendix 1 for a non-response analysis). The two measurements were combined using the unique respondent identifier, which was presented to the respondent in the announcement letter. Table 1 presents the study population at both T0 and T1.

**Table 1:** Sample characteristics

Variable	Total sample T0 (*N* = 4719)		Total sample T1 (*N* = 438)	
	*n*	%	*n*	%
*Job title of respondent*				
Director/owner	1932	40.9	173	39.5
General management	660	14.0	66	15.1
HR manager	1411	29.9	154	35.2
Other	716	15.2	45	10.3
*Number of employees*				
2–4	1292	27.4	71	16.2
5–9	628	13.3	108	24.7
10–49	964	20.4	127	29.0
50–99	1044	22.1	77	17.6
100+	791	16.8	55	12.6
Sector				
Profit	3777	80.0	340	77.6
(Semi)public	677	14.4	56	12.8
Non-profit	265	5.6	42	9.6
*Vulnerable workers hired in last 2 years*				
Yes	1651	35.0	168	38.4
No	2850	60.4	260	59.4
Uncertain	218	4.6	10	2.3
*Organizations employing certain groups of vulnerable workers*				
Mentally disabled	760	16.1	77	17.6
Psychologically vulnerable	1057	22.4	118	26.9
Physically disabled	996	21.1	112	25.6
Low-educated/learning-disabled	1109	23.5	107	24.4
Long-term unemployed	811	17.2	78	17.8
Refugees	367	7.8	34	7.8
Migrant	796	16.9	77	17.6
Actual employment of vulnerable workers^1^				
At least one vulnerable worker	–	–	189	43.2
No vulnerable workers/I do not know	–	–	239	54.6
I do not know	–	–	10	2.2
*Intended hiring of vulnerable workers in next 12 months*^1^				
Yes	–	–	115	26.3
No	–	–	207	47.3
I do not know			116	26.5
	*M*	SD	*M*	SD
*Number of vulnerable employees employed*				
Mentally disabled	0.63	6.07	1.52	14.11
Psychologically vulnerable	0.93	7.76	1.89	14.97
Physically disabled	0.63	5.74	1.47	14.12
Low-educated/learning-disabled	1.08	7.27	1.91	17.66
Long-term unemployed	0.56	3.32	0.38	1.67
Refugees	0.19	1.20	0.16	0.82
Migrant	1.65	30.14	1.36	6.93

*n* sample size, *M* mean, *SD* standard deviation

^1^Dependent variables were only measured at T1

### Measures

**Organizational characteristics** were measured at T0 with single items about sector (i.e., is the organization non-profit, profit, or (semi-)public), size of the organization (i.e., how many employees does the organization employ in total), and hiring of vulnerable workers (i.e., did the organization employ one or more vulnerable workers at T0). These variables were used as control variables in the multivariate logistic regressions.

**Social legitimacy practices** were measured at T0 and T1 with five dichotomous items, asking whether the organization explicitly mentioned (the inclusion of) vulnerable workers in their organizational mission statement**,** and whether the organization used one of the following specialized recruitment practices, specifically aimed at the recruitment of people with a distance to the labor market: (1) job creation, (2) internships, (3) hiring/seconding through an external party, or (4) collaborating with other employers to recruit vulnerable groups.

**Economic rationality practices** were measured at T0 with five dichotomous items, asking whether the organization made use of one of the following financial support measures to hire people with a distance to the labor market: (1) no-risk policy, (2) reimbursement for workplace adaptations, (2) reimbursement for the job coach, (3) unpaid trial placements of three months or (4) wage arrangements.

**Employee well-being practices** were measured at T0 with five dichotomous items, asking whether the organization applied practices to improve the sustainable employment of all employees, relating to (1) flexibility in workhours on an individual basis, (2) stimulating development of employees, (3) job adaptation or rotation, (4) retraining employees for another job or other tasks and (5) adapting the workplace.

The two outcome measures were included at T1, asking the respondent, (1) whether the organization was currently employing one or more vulnerable workers (0 = no vulnerable workers employed at T1, 1 = at least one vulnerable worker employer at T1) and 2) whether the organization had explicit plans to hire more vulnerable workers in the upcoming year (0 = organization has no hiring plans for the next 12 months, 1 = organization has concrete hiring plans for the next 12 months). Details on item formulations are presented in Appendix 2.

### Strategy of Analysis

Firstly, a descriptive analysis was performed to describe the sample in terms of application of strategic HRM practices, size of the organization and sector. Mean difference scores between size of the organization and sector were estimated using Chi-square mean difference scores. Secondly, univariate and multivariate logistic regression models were used to estimate the relation between the application of the HRM practices at T0, and the outcome measures actual employment and intended future hiring at T1. All analyses were adjusted for hiring vulnerable groups at baseline, and multivariate analyses were adjusted for sector and the number of employees at T0 as well. The odds ratios represent the odds that an organization employed a vulnerable worker at T1, given the application of a certain HRM practices at T0, while controlling for the employment of vulnerable workers at baseline. All odds ratios presented in this study have a 95% confidence interval. All analyses were performed using SPSS28.

## Results

### Descriptive Analyses

Table 1 presents the characteristics of the 438 employers included in this study and Table 2 describes the application of HRM practices based on social legitimacy, economic rationality, and employee well-being. In total, 84.5% of the employers applied one or more of the HRM practices that were included in this study. Most practices were reported less often by smaller organizations and in the profit sector.

**Table 2:** Application of strategic HRM practices and mean differences for organizational size and sector at T0

			Total sample (*N* = 4719)	Organizational size					Sector		
				a. 2–4 employees (*n* = 964)	b. 5–9 employees (*n* = 1,044)	c. 10–49 employees (*n* = 1,292)	d. 50–99 employees (*n* = 791)	e. > 100 employees (*n* = 628)	a. profit (*n* = 265)	b. (semi-) public (*n* = 677)	c. non-profit (*n* = 3,777)
Social Legitimacy practices	1	Vulnerable workers in mission	18.2%	8.2%	12.6%	19.2%	27.2%	29.8%	15.1%^b,c^	30.4%^a^	32.1%^a^
	2	Job creation	13.3%	5.7%^c,d,e^	6.6%^c,d,e^	12.2%^a,b,d,e^	20.6%^a,b,c,e^	28.8%^a,b,c,d^	11.0%^b,c^	23.0%^a^	21.1%^a^
	3	Internship(s)	45.1%	25.6%^a,b,c,d^	37.5%^a,c,d,e^	47.1%^a,b,d,e^	61.1%^a,b,c^	63.7%^a,b,c^	42.0%^b,c^	57.5%^a^	58.1%^a^
	4	Hiring/seconding through an external party	14.9%	3.9%^c,d,e^	7.4%^c,d,e^	14.1%^a,b,d,e^	26.3%^a,b,c^	31.4%^a,b,c^	13.8%^b^	20.2%^a^	16.6%
	5	Collaborating with employers	5.7%	2.8% ^d,e^	3.5% ^d,e^	4.5% ^d,e^	7.8%^a,b,c,e^	13.2%^a,b,c,d^	4.4%^b,c^	10.5%^a^	10.9%^a^
Economic rationality practices	6	No-risk policy	20.9%	6.5%^c.d.e^	11.1%^c,d,e^	19.3%^a,b,d,e^	33.0%^a,b,c,e^	47.0%^a,b,c,d^	19.2%^b^	28.8%^a^	24.5%
	7	Reimbursement for workplace adaptations	7.6%	2.9%^c,d,e^	3.6% ^d,e^	6.5%^a,d,e^	11.8%^a,b,c,e^	18.5%^a,b,c,d^	6.2%^b^	15.1%^a,c^	7.9%^b^
	8	Reimbursement for a job coach	15.9%	4.0%^c,d,e^	7.0%^c,d,e^	16.0%^a,b,d,e^	27.9%^a,b,c,e^	33.3%^a,b,c,d^	14.3%^b^	24.2%^a,c^	17.0%^b^
	9	Trial placement	19.3%	6.6%^c,d,e^	10.5%^c,d,e^	19.5%^a,b,d,e^	32.1%^a,b,c^	37.1%^a,b,c^	18.5%^b^	24.4%^a^	18.9%
	10	Wage subsidies	33.1%	11.7%^b,c,d,e^	20.5%^a,c,d,e^	33.4%^a,b,d,e^	53.9%^a,b,c^	59.9%^a,b,c^	31.4%^b^	40.8%^a^	37.0%
Employee well-being practices	11	Adapting workhours	40.5%	21.5%^b,c,d,e^	31.7%^a,c,d,e^	42.7%^a,b,d,e^	54.7%^a,b,c,e^	62.1%^a,b,c,d^	39.0%^b^	48.4%^a^	42.6%
	12	Stimulating development	23.8%	14.0%^c,d,e^	18.2%^c,d,e^	25.7%^a,b,e^	29.7%^a,b,e^	36.8%^a,b,c,d^	20.6%^b,c^	37.8%^a^	34.0%^a^
	13	Job redesign	16.0%	7.5%^c,d,e^	11.2%^c,d,e^	17.3%^a,b,d,e^	23.4%^a,b,c^	24.7%^a,b,c^	14.1%^b,c^	23.8%^a^	23.0%^a^
	14	Retraining for other job	9.9%	3.1%^c,d,e^	4.1%^c,d,e^	9.9%^a,b,d,e^	15.5%^a,b,c,e^	22.5%^a,b,c,d^	8.0%^b^	19.6%^a,c^	10.6%^b^
	15	Adapting workplace	23.0%	13.0%^c,d,e^	17.0%^c,d,e^	23.6%^a,b,d,e^	30.2%^a,b,c,e^	37.7%^a,b,c,d^	20.9%^b,c^	32.8%^a^	27.9%^a^

Significant between-group mean differences (*p* < .05), as determined by Bonferroni post-hoc analyses, are indicated with a superscript denoting the letter of the group from which the mean differs significantly

In total, 18.2% of the organizations used the social legitimacy practice of mentioning vulnerable workers in their organizational mission statement. Inclusive mission statements were more often applied in the (semi-)public and non-profit organizations compared to the profit sector. No significant differences were found for organizational size. For the inclusive recruitment practices of the social legitimacy domain, internships were most commonly applied (45.1%, other practices 5.7–13.3%). All practices were significantly more often applied in larger organizations compared to the smallest organizations. The practices were significantly less often used by employers in the profit sector, compared to the semi-public and non-profit sectors.

Among the practices in the economic rationality domain, wage subsidies were most commonly used (33.1%, other practices 7.6–20.9%). Small organizations (2–4 and 5–9 employees) applied financial practices less often compared to larger organizations. Regarding cross-sector differences, organizations in the public sector made significantly more use of all practices compared to the profit sector.

Among practices in the employee well-being domain, the most commonly applied practice was adapting work hours (40.5%, other practices 9.9–23.8%). HRM practices specifically aimed at vulnerable workers, such as retraining employees, were applied less often (9.9%) compared to practices aimed at general working populations (e.g., adapting work hours). There were several significant between-group differences, when comparing the application of employee well-being practices by organizations with different sizes (e.g., all practices were significantly more often used by larger employers) and from different sectors (e.g., all practices were significantly more often used in the (semi-)public sector).

### The Effects of Strategic HRM Practices

Table 3 shows the results of the univariate and multivariate logistic regression models that were used to explore the relation between the application of the HRM practices at T0, and the outcome measures actual employment and intended future hiring at T1.

**Table 3:** Results of logistic regression modeling of the probability of employing vulnerable workers or intentions regarding hiring vulnerable workers (*N* = 438)

Strategic HRM practices (T0)			Actual employment of vulnerable workers (T1)		Intended hiring of vulnerable workers in next 12 months (T1)	
			Univariate odds ratio (95% CI)	Multivariate odds ratio (95% CI)	Univariate odds ratio (95% CI)	Multivariate odds ratio (95% CI)
Social legitimacy practices	1	Vulnerable workers in mission	*2.515 (1.431–4.419)*	*2.587 (1.456–4.597)*	*3.198 (1.921–5.324)*	*3.358 (1.981–5.693)*
	2	Job creation	*3.260 (1.674–6.350)*	*3.080 (1.563–6.071)*	*3.920 (2.233–6.883)*	*3.621 (2.040–6.427)*
	3	Internship(s)	*1.715 (1.073–2.739)*	*1.785 (1.105–2.882)*	*3.405 (2.042–5.677)*	*3.399 (2.015–5.733)*
	4	Hiring/seconding through an external party	1.165 (0.670*–*2.026)	1.122 (0.640*–*1.966)	*1.820 (1.075–3.082)*	*1.777 (1.039–3.040)*
	5	Collaborating with employers	1.256 (0.564*–*2.798)	1.156 (0.511*–*2.613)	1.821 (0.869*–*3.817)	1.689 (0.791*–*3.609)
Economic rationality practices	6	No-risk policy	*3.038 (1.693–5.450)*	*2.995 (1.653–5.429)*	*2.961 (1.769–4.955)*	*2.923 (1.720–4.966)*
	7	Reimbursement for workplace adaptations	*3.989 (1.419–11.209)*	*3.830 (1.344–10.917)*	*3.676 (1.676–8.064)*	*3.619 (1.626–8.056)*
	8	Reimbursement for a job coach	*4.675 (2.350–9.303)*	*4.616 (2.289–9.308)*	*2.697 (1.572–4.626)*	*2.595 (1.492–4.511)*
	9	Trial placement	*3.682 (2.006–6.758)*	*3.831 (2.072–7.085)*	*2.609 (1.559–4.367)*	*2.751 (1.492–4.669)*
	10	Wage subsidies	*3.794 (2.302–6.253)*	*3.836 (2.295–6.412)*	*2.402 (1.476–3.909)*	*2.366 (1.433–3.907)*
Employee well-being practices	11	Adapting workhours	1.555 (0.984*–*2.457)	1.483 (0.919*–*2.393)	1.383 (0.877*–*2.182)	1.216 (0.753*–*1.963)
	12	Stimulating development	0.767 (0.462*–*1.276)	0.741 (0.441*–*1.242)	1.367 (0.834*–*2.241)	1.293 (0.780*–*2.142)
	13	Job redesign	1.133 (0.660*–*1.943)	0.991 (0.565–1.737)	*1.764 (1.056–2.948)*	1.511 (0.884–2.580)
	14	Retraining for other job	1.011 (0.458*–*2.230)	0.995 (0.448*–*2.209)	1.366 (0.638*–*2.926)	1.266 (0.586*–*2.738)
	15	Adapting workplace	1.194 (0.705*–*2.020)	1.183 (0.695*–*2.015)	0.917 (0.545*–*1.544)	0.884 (0.520*–*1.502)

Rows in this table present univariate logistic regression analyses of one strategic HRM practice with each outcome variables, adjusted for employment of vulnerable workers at baseline, and multivariate logistic regression analyses adjusted for the number of employees in the organization, sector (0 = profit or semi-public, 1 = non-profit) and employment of vulnerable workers at baseline; CI = confidence interval; cells reporting statistically significant relations are italics

First, we explored whether social legitimacy practices at baseline were associated with the employment of vulnerable workers and hiring intention for the upcoming year at follow-up. The results of the multivariate regression analyses showed that an inclusive mission statement predicted employment of vulnerable workers (OR = 2.59, 95% CI [1.46–4.60]) and hiring plans for the upcoming year (OR = 3.36, 95% CI [1.98–5.69]). Furthermore, the results of the multivariate analyses show that employers, who engaged in job creation or internships were more likely to employ a vulnerable worker at follow-up (job creation: OR = 3.08, 95% CI [1.56–6.07]; internships: OR = 1.79, 95% CI [1.11–2.88]) and to have hiring intention at follow-up (job creation: OR = 3.62, 95% CI [2.04–6.423]; internships: OR = 3.40, 95% CI [2.02–5.73]). No significant relationship was found between engaging in hiring/seconding through an external partner and the employment of vulnerable workers at follow-up (OR = 1.12, 95% CI [0.64–1.97]). Still, the organizations that hired or seconded through an external partner were significantly more likely to have hiring plans for the upcoming year at follow-up (OR = 1.78, 95% CI [1.04–3.04]). No support was found for the recruitment practice of collaborating with other employers to contribute to employment of vulnerable workers.

Secondly, we explored whether the use of economic rationality practices at T0 was associated with the employment of vulnerable workers and future hiring intention at T1. The results of the multivariate regression in Table 3 showed that all practices in this domain significantly predicted employment of vulnerable workers at follow-up (no-risk policy: OR = 3.00, 95% CI [1.65–5.43]; reimbursement for workplace adaptations: OR = 3.83, 95% CI [1.34–10.92]; reimbursement for a job coach: OR = 4.62, 95% CI [2.29–9.31]; trial placement: OR = 3.83, 95% CI [2.07–7.09]; wage subsidies: OR = 3.84, 95% CI [2.30–6.41]). Furthermore, all practices in this domain were significantly related with the intention to hire vulnerable workers in the next 12 months (no-risk policy: OR = 2.92, 95% CI [1.72–4.97]; reimbursement for workplace adaptations: OR = 3.62, 95% CI [1.63–8.06]; reimbursement for a job coach: OR = 2.60, 95% CI [1.49–4.51]; trial placement: OR = 2.75, 95% CI [1.49–4.67]; wage subsidies: OR = 2.37, 95% CI [1.43–3.91]).

Lastly, we explored whether employee well-being practices at T0 were associated with the employment of vulnerable workers and hiring plans at T1. Limited support was found for this, since no significant relations were found between the practices in this domain (adapting work hours, stimulating development, job redesign, retraining an individual for another job, or adapting the workplace) and the outcome measures, after correcting for organizational size, sector and employment of vulnerable workers at baseline.

## Discussion

The aim of this study was to investigate to what extent different types of strategic HRM practices are related to the actual employment of vulnerable workers and the intention to hire vulnerable workers nine months later. Our results showed that employers who applied strategic HRM practices based on social legitimacy and economic rationality were significantly more likely to employ vulnerable workers at follow-up. With this finding, we show the effects of strategic HRM practices on both the intended and actual employment of those who have a vulnerable position on the labor market, such people with disabilities, but also people that are long-term unemployed or refugees. Thereby, our study provides evidence that specialized HRM practices aimed at vulnerable workers are not solely a method of window-dressing but make a significant impact on the inclusion of vulnerable groups on the labor market.

Concerning practices based on social legitimacy, previous research has shown that organizations may hire vulnerable workers if they believe that this may result in competitive advantage and an improved ‘employer brand’ [16]. HRM practices based on social legitimacy, such as an inclusive mission statement, may support these goals. Further, previous research described challenges related to the recruitment process and hiring process of vulnerable groups of workers [31]. Inclusive recruitment practices of the social legitimacy domain, aimed at job creation and internships, may contribute to overcoming these challenges. Regarding practices based on economic rationality, our results align with previous research that shows that many employers experience barriers related to costs [31]. Employers may overcome these cost-related barriers by applying economic rationality practices, such as making use of reimbursements or subsidies.

Employers that used practices based on social legitimacy and economic rationality were also significantly more likely to intend to hire vulnerable workers at follow-up, compared to organizations that did not use these practices. This finding aligns with the Integrative Model of Behavioral Prediction [40], which has previously been applied to study inclusive employer behavior [e.g., 31, 41]. According to this theoretical model, strategic HRM practices increase the organizational resources that are needed to employ vulnerable workers, while also increasing the employer’s self-efficacy (i.e., the belief that the organization can successfully hire vulnerable workers). This self-efficacy, in turn, may also positively influence the intention to hire vulnerable workers in the future.

Contrary to our expectations, HRM practices that target employee well-being were not related to the employment of vulnerable workers and the intention to hire vulnerable workers at follow-up. This finding is not in line with previous research that suggests that changes to work tasks and arrangements initiated by the employee (job crafting) or by the employer (job carving) [42], as well as workplace adaptations may help to promote the inclusion of vulnerable workers [43]. A potential explanation for this unexpected result may be that, while the economic rationality and social legitimacy practices were all measured as practices that are specifically aimed at vulnerable groups, employee well-being practices were conceptualized as practices that are aimed at the general population of employees, including both vulnerable and non-vulnerable workers (see Appendix 2). This may indicate that the inclusive employment of vulnerable groups requires strategic HRM practices that are specifically designed to support these workers. An additional explanation could be that, in contrast to the present study, practices in the well-being domain such as job crafting, were previously studied on the individual-level in relation to employee-level outcomes (e.g., well-being or job embeddedness), for instance in unemployed individuals [44], migrant workers [45] or workers with disabilities [46]. Therefore, future research may benefit from studying the practices relating to the employee well-being domain in a multi-level perspective, taking into account the intended, actual and perceived nature of these practices [47]. Also, it seems likely that HRM practices that target employee well-being (e.g., stimulation of development, retraining for a job or job redesign) may be particularly valuable to stimulate the sustainable employment of vulnerable workers, as these practices may guarantee the employee’s well-being and employability over time. Therefore, these practices might be stronger predictors of long-term well-being and employability of vulnerable workers compared to the hiring of vulnerable workers.

### Limitations and Future Research Suggestions

This study is subject to several limitations. First, the NEWS-2021 questionnaire is filled in by a single respondent per organization (i.e., directors or HR/general managers). Particularly in larger organizations, these respondents may not be fully informed about all relevant organizational practices. This may have influenced our findings. Second, organizations reporting night work at baseline could not be included in this study. Additional analyses showed these organizations significantly more often offered inclusive HRM practices and employed and hired vulnerable workers. Further research is needed to explore whether the relation between these variables differ between organizations with or without nightshifts. Third, the follow-up period of nine months in this study was relatively short, making it impossible to draw conclusions on the effects of HRM practices over a longer period of time. Fourth, in this study we explored whether the use of strategic HRM practices was related to the (continued) employment of vulnerable groups within the organization, as well as future hiring intentions. It could be argued, however, that these relationships may also be reversed, e.g., organizations may start to make certain workplace adaptations only after they have hired vulnerable who have a need for these adaptations. Therefore, future research could address this issue. Fifth, the descriptions of HRM practices and vulnerable worker groups in the questionnaire may not have been clear to all participants (see Appendix 2). We do not know how this influenced our findings. Sixth, this study did not include a measure on tenure of (vulnerable) employees, and hence, we were not able to study the relation between HRM practices and individual-level tenure of participants over time. We suggest that future research includes measurements on vulnerable workers’ turnover and tenure within the organization. Seventh, because the data collection was part of a large-scale monitoring study of the Dutch labor market (i.e., NEWS), most items included in this questionnaire were of binary nature. Future research could benefit from including validated, multi-item and Likert-scales [e.g., 19]. Eight, although we included a wide variety of organizational practices, we recommend future researcher to include other potentially relevant (HRM) practices, related to senior management commitment, organizational culture, and monitoring of the (perceived) inclusion of vulnerable workers as well [15]. Further, we recommend future research to study whether the relationship between inclusive HRM practices and employment of vulnerable groups differs between different groups of vulnerable workers, e.g., people with learning disabilities, people with physical disabilities, people with a migration background, refugees. Finally, since countries differ in the availability of (financial) practices [48], future research is recommended to study whether our findings hold in different national contexts.

### Implications

Our findings provide important insights for employers, policymakers, rehabilitation experts and employees. Firstly, for employers, our study shows that various strategic HRM practices contribute to successful hiring of vulnerable workers. By showing this, our study extends previous research that (1) focused only on the application of HRM practices without investigating the relation with actual employment of vulnerable workers [e.g., 49], (2) addressed only people with disabilities but not other groups of vulnerable workers [e.g., 18, 19] or (3) studied overarching groups of inclusive HRM practices, without specifying the effects of individual practices [e.g., 31]. By showing the effects of different, individual HRM practices for the employment and intended employment of vulnerable workers, our study helps employers to create inclusive and strategic HRM systems that help to create inclusive workplaces for vulnerable workers.

For policymakers, an important finding of this study is the positive influence of financial support practices, offered by the government, on inclusive employer behavior. Our study shows that these practices were used significantly more often by larger employers compared to smaller employers. This aligns with previous research on the Dutch labor market, which shows that 46% of employers with fewer than 20 employees are not familiar with no-risk policies, compared to 21% of employers with more than 100 employees [50]. This may be because these financial arrangements require specific expertise and knowledge of governmental arrangements, which may be present in larger companies with extensive HR departments and may be lacking in smaller companies that do not have this HR expertise. Therefore, we recommend making these policy measures more accessible for smaller organizations.

The findings of this study are also valuable to rehabilitation experts that educate and support employees and employers in reintegration. Rehabilitation experts play an important role in bringing the effective HRM practices that were found in our study to the attention of the employer and to support them in the implementation of these HRM practices. Further, this study shows that rehabilitation experts need to educate and support employers beyond measures related to hiring of vulnerable workers that were already described in the rehabilitation literature [51], searching for the right fit between HRM practices and strategic organizational goals and strengths relation to social legitimacy, economic rationality and employee well-being.

Together, our findings help to professionalize the demand-side efforts of employers to improve the labor market inclusion of various vulnerable groups of workers by showing which strategic HRM practices may contribute to inclusive employer behavior.

## Supplementary Information

Below is the link to the electronic supplementary material.

Supplementary file1 (DOCX 60 KB)

Supplementary file2 (DOCX 61 KB)

## Data Availability

The datasets generated during and/or analysed during the current study are available in a benchmarking tool on: https://www.monitorarbeid.tno.nl/nl-nl/onderzoeken/wea/.
