# Supplementary material for: The Relationship Between Strategic Human Resource Management Practices and the Employment of Vulnerable Workers: A Two-Wave Study Among Employers
Source: J Occup Rehabil. 2024 May 4;35(2):294–305. doi: 10.1007/s10926-024-10197-9 (PMC12089176; doi:10.1007/s10926-024-10197-9)
Supplement: Supplementary file 1 — Supplementary file1 (DOCX 60 KB) [file 10926_2024_10197_MOESM1_ESM.docx]

# Appendix 1 Non-response analysis

| Table A1. Non-response Analysis | | | | | | |
| --- | --- | --- | --- | --- | --- | --- |
|  | | | T0 measurement of non-responders  (*N* = 556) | T0 measurement of total sample T1  (*N* = 438) | χ^2^ value | *p*  value |
| Social Legitimacy practices | Vulnerable workers in mission at T0 | | 18.3% | 21.2% | 1.295 | .255 |
|  | Job creation | | 15.1% | 15.8% | .078 | .779 |
|  | Internship(s) | | 50.5% | 51.1% | .036 | .851 |
|  | Hiring/seconding through an external party | | 17.3% | 18.7% | .353 | .552 |
|  | Collaborating with employers | | 6.7% | 8.0% | .651 | .420 |
| Economic rationality  practices | No-risk policy | | 21.9% | 20.8% | .198 | .656 |
|  | Reimbursement for workplace adaptations | | 7.9% | 7.1% | .245 | .620 |
|  | Reimbursement for a job coach | | 17.1% | 17.4% | .012 | .912 |
|  | Trial placement | | 17.4% | 20.3% | 1.330 | .249 |
|  | Wage subsidies | | 34.5% | 34.9% | .017 | .896 |
| Employee  well-being  practices | Adapting workhours | | 45.1% | 48.6% | 1.196 | .274 |
|  | Stimulating development | | 26.8% | 27.9% | .138 | .711 |
|  | Job redesign | | 17.6% | 20.8% | 1.579 | .209 |
|  | Retraining for other job | | 8.6% | 7.3% | .583 | .445 |
|  | Adapting workplace | | 24.5% | 24.2% | .009 | .925 |
| Hiring  practices | Hired vulnerable workers in the past two years | | 37.2% | 38.4% | .628 | .730 |
|  | Currently employing at least one vulnerable worker | | 59.5% | 62.3% | .804 | .370 |
| Covariates | Organizational size^1^ | | - | - | 6.465 | .167 |
|  |  | 2 – 4 employees | 17.3% | 16.2% | .196 | .658 |
|  |  | 5 – 9 employees | 22.6% | 24.7% | .542 | .462 |
|  |  | 10 – 49 employees | 31.5% | 29.0% | .712 | .399 |
|  |  | 50 – 99 employees | 17.3% | 17.6% | .017 | .897 |
|  |  | > 100 employees | 11.3% | 12.5% | .352 | .553 |
|  | Organizational sector^2^ | | 5.6% | 9.6% | 5.800 | .016 |
| *Note.* ^1^ organizational size was included in the final analysis as a categorical variable (1 = 2-4 employees; 2 = 5-9 employees; 3 = 10-49 employees; 4 = 50-99 employees; 5 = 100+ employees); ^2^ sector was included in the final analyses as a binary variable (0 = profit or (semi-)public sector; 1 = non-profit sector); The hiring practices included in the non-response analysis were measured at T0 and are different from the dependent variables included in the final analyses, which were measured at T1; cells reporting statistically significant relations are colored grey | | | | | | |
