# Supplementary material for: The Relationship Between Strategic Human Resource Management Practices and the Employment of Vulnerable Workers: A Two-Wave Study Among Employers
Source: J Occup Rehabil. 2024 May 4;35(2):294–305. doi: 10.1007/s10926-024-10197-9 (PMC12089176; doi:10.1007/s10926-024-10197-9)
Supplement: Supplementary file 2 — Supplementary file2 (DOCX 61 KB) [file 10926_2024_10197_MOESM2_ESM.docx]

# Appendix 2 Item Formulations

| Table A2. Item Formulations (translated from Dutch) | | |  |  |
| --- | --- | --- | --- | --- |
| **Item label** | | **Item formulation** |  |  |
| **Social legitimacy practices (IV; T0)**  **🡪** *Added as binary variables (0 = practice was marked by participant, 1 = practice was not marked by participant)* | |  |  |  |
|  | *Vulnerable workers in mission* | Is engaging "vulnerable groups" explicitly part of the mission statement of your facility?   - Yes - No - I do not know |  |  |
|  | | What kind of actions are you taking (or do you specifically plan to take) to hire people with disabilities?  [multiple answers possible] |  |  |
|  | *Job creation* | - Creation of jobs in new contracts/procurement (by using, for example, Social Return) - Creation of new jobs from existing jobs (by using, for example, job creation/job differentiation/job carving) |  |  |
|  | *Internship(s)* | - Offer a work experience place, internship, apprenticeship project |  |  |
|  | *Hiring/seconding through an external party* | - Hiring/secondment (via an external party) |  |  |
|  | *Collaborating with employers* | - Work with other employers in the region to look at the supply of work/tasks suitable for "vulnerable groups" (using, for example, shared employment/regional labor or task pools) |  |  |
| **Financial practices (IV; T0)**  **🡪** *Added as binary variables (0 = practice was marked by participant, 1 = practice was not marked by participant)* | | Has your establishment used one or more of the following regulations when hiring partially disabled persons?  [multiple answers possible] |  |  |
|  | *No-risk policy* | - No-risk policy for sickness and disability (in the first 5 years after hiring) |  |  |
|  | *Reimbursement for workplace adaptations* | - Reimbursement for adaptation of the workplace |  |  |
|  | *Reimbursement for a job coach* | - Job coach: reimbursement for additional guidance at work |  |  |
|  | *Trial placement* | - Trial placement of up to 3 months without having to pay salary |  |  |
|  | *Wage subsidies* | - Wage dispensation for the young disabled - Wage cost benefits - Wage subsidy from municipality |  |  |
| Table A2. *Continued* | | |  |  |
| **Employee well-being (IV; T0)**  **🡪** *Added as binary variables (0 = practice was marked by participant, 1 = practice was not marked by participant)* | | What provisions/measures are in place at your facility to allow all employees to work longer?  [multiple answers possible] |  |  |
|  | *Adapting workhours* | - Shorter working week/adjustment of working hours on an individual basis |  |  |
|  | *Stimulating development* | - Encourage training and/or course participation |  |  |
|  | *Job redesign* | - Adaptation of job tasks (job crafting) |  | |
|  | *Retraining for other job* | - Retraining to another job/role |  | |
|  | *Adapting workplace* | - Adaptation of the workplace |  | |
| **Actual employment of vulnerable workers (DV; T1)**  **🡪**  *Added as a binary dependent variable (0 = no or I do not know, 1= yes)* | | Does your facility currently employ individuals from one or more vulnerable groups, such as the long-term unemployed, high school dropouts and young or partially disabled people?   - Yes - No - I do not know |  |  |
| **Intended hiring of vulnerable workers (DV; T1)**  **🡪**  *Added as a binary dependent variable (0 = no or I do not know, 1= yes)* | | In the next 12 months, does your facility intentionally plan to hire individuals from one or more vulnerable groups, such as the long-term unemployed, high school dropouts and young or partially disabled people?   - Yes - No - I do not know |  |  |
| **Number of employees in the organization (C; T0)**  ***🡪*** *Added as a continuous control variable* | | - What is the total number of employees within your facility? [open ended question] |  |  |
| **Employment of vulnerable workers at baseline (C; T0)**  **🡪** *Added as a binary control variable (0 = no employees from any group; 1 = at least one employee of at least one of these groups)* | | Approximately how many employees within your facility are a part of the following “vulnerable groups”? [open ended question for each group below]   - People with (mild) intellectual disabilities - People who are mentally vulnerable - People with physical disabilities - People with insufficient education/learning disabilities - People who have been long-term unemployed - Refugees - People with a migration background |  |  |
| **Sector (C; T0)  🡪** *Added as a binary control variable (0 = business, public or semi-public; 1 = private, non-profit)* | | How would you describe your facility?   - Business (profit-oriented/for profit) - Public or semi-public (government organization, independent governing body, research/educational institution, etc.) - Private, non-profit (charitable foundation, social enterprise, etc.). |  |  |
| *Note*. IV = independent variable; DV = dependent variable; C = control variable. | | |  |  |
